# A New Model of Short-Term Motor Adaptation

**DOI:** 10.1371/journal.pbio.0040205

**Published:** 2006-05-23

**Authors:** Liza Gross

Starting at around three months old, children can finally reach for the countless toys their parents have been dangling before them since birth. These attempts often involve a good deal of flailing about, as motor skills, like anything else, require cultivation. Motor control depends on executing the proper musculoskeletal force to reach the desired object.

Prior learning facilitates motor control (all that flailing serves a purpose), which is aided by a fundamental feature of memory, called savings. When a novel response to a stimulus is learned in one set of trials, then “washed out” in an unlearning phase, subsequent relearning proceeds faster. Neuroscientists have been puzzled by savings and other features such as interference and rapid unlearning reported in adaptation studies because standard models of short-term motor adaptation couldn't explain them. But now Maurice Smith, Ali Ghazizadeh, and Reza Shadmehr have solved this puzzle by combining experimental and computational approaches, and show that two adaptive brain components with different learning rates and retention capacities can account for these seemingly disparate properties of learning.

In a recent study of savings in eye-saccade adaptation (saccades are rapid eye movements that shift gaze direction) in monkeys, Yoshiko Kojima et al. explained the savings they observed with a model in which two distinct mechanisms increased or decreased the amplitude gain of saccades based on a target's direction. This “two-state, gain-specific” model explained the savings and washout effects the researchers had seen. But Kojima et al. found that when adaptation was extinguished, and then time passed with the monkey receiving no feedback on its behavior, there was a sudden recovery of the animal's saccade gains toward the initial learning state. This spontaneous recovery could not be explained by the gain-specific model. Nor, Smith et al. explain, can the gain-specific model explain a property of learning called anterograde interference, in which initial motor learning is faster than subsequent adaptation to an opposing task.

Smith et al. focused on the spontaneous recovery phenomenon: since behavior changed even when there was no error feedback as a guide, they reasoned that motor error could not be the only factor affecting motor learning and that passage of time must have also played a role. They considered a system in which two states both learned from error: one learned rapidly but tended to quickly forget, while the other learned slowly and tended to remember.

To test their hypothesis, they first designed a variation on a standard paradigm for studying how humans learn to compensate for an imposed force while reaching for a target. Participants held a joystick-like, robot-controlled “manipulandum,” which measures hand position and velocity, and applies well-controlled counter forces to the hand. Participants' hand position was visible on a computer monitor in front of them, and they were told to quickly reach toward small circular targets spaced ten centimeters apart along a horizontal plane. After practicing without the robot motors, participants adapted their reaching movements to a force-field perturbation pushing their hand in one direction; then learning was extinguished by adaptation to an opposing force field. Error feedback was then removed with an “error clamp” that forces the hand along a straight line to eliminate lateral errors during movement. Even though participants were kept from experiencing lateral errors, the manipulandum recorded their lateral force resistance to the error clamp, so the researchers could infer participants' motor plans and track the evolution of learned changes in motor output.

As predicted by the model, the observed spontaneous recovery suggested that two different learning states—one with a fast time scale and another with a slower time scale—combined to produce motor output. Rebound occurs because the fast-learning module decays quickly during the no-feedback block, but the slow-learning module decays gradually, allowing a transient recovery of motor output.

Altogether, these results argue for a learning system based on two time courses and explain a wide range of phenomena associated with short-term motor adaptation. The multirate model accounts for anterograde interference, by showing that the slow learning component is initially biased against learning the second adaptation. And it explains “rapid unlearning”—in which the rate of de-adaptation after a period of learning can be much faster than initial learning—and additionally predicts that this de-adaptation is fastest after short adaptation blocks, then declines as the amount of training in the initial adaptation increases.

It may well be that different functional units in the brain learn motor control at different rates—a possibility that researchers can explore with the help of this unifying model of motor adaptation. And by combining behavioral experiments with targeted brain lesions and functional imaging in the cerebellum—which is required for normal motor adaptation—researchers can investigate what regions of the brain control these learning modules.

## 

**Figure:**
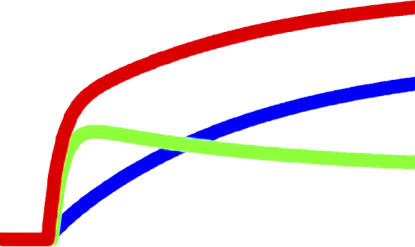
Two learning processes with distinct time courses contribute significantly to short-term motor skill acquisition, and the interactions between these processes explain a host of puzzling phenomena in motor adaptation.

